# Antibiotic Susceptibility Patterns and Virulence Profiles of Classical and Hypervirulent *Klebsiella pneumoniae* Strains Isolated from Clinical Samples in Khyber Pakhtunkhwa, Pakistan

**DOI:** 10.3390/pathogens14010079

**Published:** 2025-01-15

**Authors:** Azra, Taj Ali Khan, Ihtisham Ul Haq, Woranich Hinthong, Susana Campino, Aisha Gohar, Noman Khan, Muhammad Kashif, Ihsan Ullah, Taane G. Clark

**Affiliations:** 1 Institute of Pathology and Diagnostic Medicines, IPDM, Khyber Medical University Peshawar, Peshawar 25000, Pakistan; azra.ibms@kmu.edu.pk (A.); tajalikhan.ibms@kmu.edu.pk (T.A.K.); 2 Emerging Pathogens Institute, University of Florida, Gainesville, FL 32611, USA; 3 Department of Physical Chemistry and Technology of Polymers, Silesian University of Technology, 44-100 Gliwice, Poland; ihaq@polsl.pl; 4 Joint Doctoral School, Silesian University of Technology, 44-100 Gliwice, Poland; 5 Postgraduate Program in Technological Innovation, Federal University of Minas Gerais, Belo Horizonte 31270-901, Brazil; 6 Faculty of Infectious and Tropical Diseases, London School of Hygiene and Tropical Medicine, London WC1E 7HT, UK; woranich.hinthong@lshtm.ac.uk (W.H.); susana.campino@lshtm.ac.uk (S.C.); 7 Faculty of Epidemiology and Population Health, School of Hygiene and Tropical Medicine, London WC1E 7HT, UK; 8 Microbiology Department, Bacha Khan Medical College Mardan, Mardan 23200, Pakistan; ashekhan706@gmail.com; 9 Al Rasheed Hospital & Kidney Center, Opposite Gilani Mart, Maneshra Road, Abbottabad 22020, Pakistan; nomanrozii@gmail.com; 10 Public Health Reference Laboratory, Khyber Medical University Peshawar, Peshawar 25000, Pakistan

**Keywords:** antibiotic resistance, virulence, biofilm, *K. pneumoniae*, hypervirulent, multidrug resistant, carbapenem resistant, public health

## Abstract

The emergence of hypervirulent and carbapenem-resistant hypermucoviscous *Klebsiella pneumoniae* strains presents a significant public health challenge due to their increased virulence and resistance to multiple antibiotics. This study evaluates the antibiotic susceptibility patterns and virulence profiles of classical and hypervirulent *K. pneumoniae* strains isolated from various clinical samples. A total of 500 clinical samples were collected from patients at the Mardan Medical Complex and Ayub Medical Complex in KPK between July 2022 and June 2024. Among these, 64 *K. pneumoniae* strains were isolated and subsequently subjected to antimicrobial susceptibility testing (AST) and phenotypic virulence detection. Among the 64 isolates, 21 (32.8%) exhibited hypermucoviscosity, a characteristic associated with increased pathogenicity. Hemagglutination was observed in 35 (54.1%) of the isolates, indicating the presence of surface adhesins that facilitate bacterial adherence to host tissues. A high prevalence of biofilm formation was noted, with 54 (84%) isolates capable of forming biofilms, which are known to protect bacteria from antibiotics and the host immune response. Most isolates (59/64, 92.1%) were resistant against ampicillin, highlighting its limited efficacy against these strains. Conversely, the lowest resistance was observed for tigecycline, with only 15% (10/64) of the isolates showing resistance, indicating its potential utility as a treatment option. The study also found that 38 (59.3%) of the isolates were extended-spectrum beta-lactamase (ESBL) producers, 42 (65.6%) were multidrug-resistant (MDR), 32 (50%) were extensively drug-resistant (XDR), and 13 (20.3%) were resistant to carbapenems. The genetic study revealed biofilm producer and enhancer genes (*mrkD*, *pgaABCD*, *fimH*, *treC*, *wzc*, *pilQ*, and *luxS*) mainly in the hypervirulent strains. These hypervirulent strains also show a high number of resistance genes. The findings of this study underscore the critical need for the active surveillance of antimicrobial resistance and virulence determinants in *K. pneumoniae*. The coexistence of high levels of antibiotic resistance and virulence factors in these isolates poses a severe threat to public health, as it can lead to difficult-to-treat infections and increased morbidity and mortality.

## 1. Introduction

*Klebsiella pneumoniae* is a Gram-negative, encapsulated, non-motile bacillus notable for its lactose-fermenting capability and facultative anaerobic metabolism. As a member of the Enterobacteriaceae family, it is a significant pathogen in hospital and community environments [1,2], causing a wide range of illnesses, including liver abscesses, pneumonia, bacteremia, urinary tract infections, and digestive system infections [1]. Using some medical equipment, including respiratory support apparatus and different kinds of catheters [3], increases the risk of *K. pneumoniae* infections. The incidence of nosocomial infections among hospitalized patients worldwide was 8.7%, and individuals undergoing surgery, the transplantation of organs, or cancer may be more vulnerable [4]. Importantly, *K. pneumoniae* is the cause of up to 10% of nosocomial infections [5]. In Singapore, the mortality rates from *K. pneumoniae* bacteremia ranged from 20 to 26 percent [6], whilst in China, *K. pneumoniae* was responsible for 11.9% of the pathogens isolated from ventilator-associated pneumonia (VAP) and intensive care unit (ICU)-acquired pneumonia [7]. Furthermore, in a multicenter clinical study covering 25 “AAA” hospitals in 14 provinces of China, carbapenem-resistant Enterobacteriaceae (CRE) caused by *K. pneumoniae* was reported to account for 73.9% of 664 clinical samples [8]. These high prevalence and mortality rates of *K. pneumoniae* infections place a significant strain on the nation’s healthcare system.

Various virulence factors that successfully thwart the host’s innate immune systems and promote the infection’s persistence are responsible for *K. pneumoniae’s* pathogenicity. The lipopolysaccharides, adhesins, siderophores, bacterial capsules, and biofilm development are essential factors determining pathogenicity [9]. The range of expressed virulence factors and the clinical symptoms of illnesses caused by *K. pneumoniae* are closely related [10]. Notably, the capsular polysaccharide protects the bacteria from the bactericidal activities of serum and expert phagocytes [11]. Furthermore, the type 1 and type 3 fimbrial adhesins encoded by the *fimH-1* and *mrkD* genes are essential for the production of biofilms, which aids in establishing infections [12]. The pathogenicity and severity of *K. pneumoniae* infections are influenced mainly by several genes, for example, regulators of the mucoid phenotype A (*rmpA*) and the microviscosity-associated gene A (*magA*) [13]. Additional genes of importance include those related to iron siderophore aerobactin production (*aero*), iron absorption (*kph*), yersiniabactin biosynthesis (*ybtS*), enterobactin biosynthesis (*entB*), and allantoin metabolism (*allS*) [13]. These genetic elements increase *K. pneumoniae’s* pathogenic potential and infection severity.

The last ten years have seen a severe increase in the incidence of *K. pneumoniae* that produces extended-spectrum β-lactamase (ESBL), which has severe implications for global public health. The generation of carbapenemases and extended-spectrum β-lactamases (ESBL) results in substantial death rates (40–50%), mainly in patients who are acutely sick and immunocompromised [14]. *K. pneumoniae* is shielded from all current antibiotics by the formation of strains that are extensively drug-resistant (XDR) and pan-drug-resistant (PDR) due to the ongoing accumulation of antibiotic-resistant genes [15,16]. The World Health Organization (WHO) has identified the rising incidence of multidrug-resistant (MDR) *K. pneumoniae* as a significant global health concern, with *OXA-48* and *NDM* amongst the most prevalent carbapenemase types, including in Saudi Arabia [17,18,19,20].

Bacteria responsible for hospital-acquired infections (HAIs) have developed resistance to multiple antibacterial drugs, underscoring the urgent need for community-wide antimicrobial resistance (AMR) monitoring [21]. AMR in *Klebsiella* spp. has become more common in Pakistan, especially in the province of Khyber Pakhtunkhwa [22,23,24]. Additionally, the concurrent presence of virulence factors and drug-resistance genes may synergistically exacerbate infection severity [25]. However, more local information is needed about the patterns of antibiotic sensitivity and the genetic underpinnings of the phenotypic pathogenicity of *K. pneumoniae* isolates from various samples. In light of this situation, the current study aimed to analyze the antibiotic susceptibility patterns and phenotypic virulence patterns of various clinically isolated *K. pneumoniae* and perform the molecular characterization of genes associated with virulence and antibiotic resistance in the identified bacterial isolates. This study also identified the relationship between an organism’s capacity to produce biofilms and its resistance to certain antibiotics through whole-genome sequencing.

## 2. Materials and Methods

### 2.1. Sample Collection

Using a random convenient sampling technique, a total of 500 cases were included in this study, with samples collected from urine, pus, wounds, blood, and other body fluids. Before sample collection and ethical approval were granted (Ref/no: KMU/IPDM/IEC/202229), all study participants were informed about the sample collection techniques. A random selection method was employed to gather the 500 samples, ensuring adherence to conventional sanitation standards and microbiological protocols [26]. The samples were aseptically inoculated onto MacConkey agar (Oxoid, UK) medium and incubated at 37 °C for 24 h. To confirm and differentiate from *Escherichia coli*, mucoid, circular, and lactose-fermenting colonies were subcultured on Eosin Methylene Blue agar (EMB) (Oxoid, UK).

### 2.2. Pathogens Identification

Biochemical tests were conducted to characterize the presumptively identified isolates. These tests included catalase, oxidase, urease, indole production, motility, methyl red, Voges-Proskauer, and citrate utilization tests [27]. Following biochemical identification, the strains were confirmed using the API 20E kit (bioMérieux SA, Lyon, France) and showed a match with reference *K. pneumoniae* catalog number 5 214 573. The confirmed isolates were then preserved in a Luria–Bertani broth medium supplemented with 40% glycerol and stored at −80 °C. For short-term maintenance, productive cultures were kept on nutrient agar at temperatures between 2 °C and 8 °C, with an average viability of four weeks.

### 2.3. Antimicrobial Susceptibility Test

Antimicrobial susceptibility testing (AST) was conducted on all isolates using the Mueller–Hinton agar (MHA) method (Oxoid, Manchester, UK) and a disc diffusion assay [28]. A 0.5 McFarland standard-turbidity suspension was prepared and applied to the MHA plate surface using a sterilized cotton swab. Fourteen antibiotic discs, including ampicillin (AMP, 10 µg), amikacin (AK, 30 µg), amoxicillin–clavulanic acid (AMC, 20/10 µg), cefotaxime (CTX, 30 µg), ceftazidime (CAZ, 30 µg), ciprofloxacin (CIP, 30 µg), cefepime (FEP, 6.5 µg), Sulbactam/Cefoperazone (SCF, 1.5 µg), piperacillin–tazobactam (TZP, 4.5 µg), Ceftazidime/avibactam (CZA, 2.5 µg), tigecycline (TGC, 0.5 µg), gentamicin (CN, 10 µg), tetracycline (TET, 30 µg), and meropenem (MEM, 10 µg) were placed on the MHA plates, which were then incubated at 37 °C for 24 h. These antibiotics are commonly used for *K. pneumoniae* treatment. The inhibition zones were measured to the nearest millimeter and interpreted according to the Clinical and Laboratory Standards Institute (CLSI M100 Ed33) guidelines [29,30]. The MDR and potential XDR strains were identified following the guidelines recommended by the European Centre for Disease Prevention and Control (ECDC) [31].

### 2.4. String Testing

The hyperviscosity (Hv) phenotype was assessed using the Modified String Test. A strain of *K. pneumoniae* was inoculated onto a sheep blood agar medium and incubated at 37 °C for 24 h. Following incubation, an inoculation loop was used to perform the string test. The Hv phenotype was indicated by forming a string more significant than 5 mm long [32].

### 2.5. Hemolysis

To evaluate the hemolytic potential of *K. pneumoniae*, 5% rabbit blood was added to a Blood Agar Base (OXOID, Hampshire, UK). A fresh culture of *K. pneumoniae* was inoculated through loop onto the blood agar plates and incubated for 24 h at 37 °C. The plates were then incubated for an additional 24 h for optimal hemolytic activity. After the complete incubation period, the hemolytic zones surrounding the *K. pneumoniae* colonies were examined to assess their hemolytic potential.

### 2.6. Hemagglutination Assays

Human Group O+ blood was used to determine hemagglutination assay; it was obtained and stored immediately at 4 °C until it was needed. The RBCs were suspended at a 3% (vol/vol) concentration in phosphate-buffered saline (PBS), which has a pH of 7.4, after being washed three times. Using McFarland turbidity standards [33], plate-grown bacterial cultures were adjusted to around 1.5 × 10^8^ bacteria per milliliter in PBS. After being collected, broth-grown cultures were suspended in PBS to equal the turbidity of plate-grown cultures after being twice washed in PBS.

On a slide, 20 µL of the bacterial solution and 20 µL of the blood cell suspension were combined, and the mixture was gently shaken by hand to perform hemagglutination. For every test, a PBS–blood cell control was used. Within ten minutes, strains that did not show signs of hemagglutination were categorized as negative hemagglutinators [34,35,36].

### 2.7. Biofilm Formation

The biofilm activity of the isolates was assessed using the tube method. To standardize the bacterial inoculum to 0.5 McFarland standards (1.5 × 10^8^ CFU/mL) [33], 50 μL of overnight culture in LB broth was diluted and transferred into tubes containing 2 mL of sterile LB broth. The tubes were then incubated for 24 h at 37 °C without agitation. The experiment included a negative control (without bacterial inoculum) and a positive control with a confirmed biofilm-producing strain. After incubation, the broth cultures were discarded, and the tubes were washed twice with PBS to remove non-adherent bacteria. The tubes were stained with 0.1% crystal violet for 30 min to visualize biofilm formation. Excess dye was decanted, and the tubes were washed with deionized water to remove unbound dye. After drying, biofilm production was evaluated based on the presence of blue staining on the inner walls of the tubes [37,38].

### 2.8. Whole-Genome Sequencing

Twelve isolates were chosen for whole-genome sequencing (WGS) based on whether they were strong biofilm producers or did not generate biofilm, as well as the antibiotic resistance pattern based on AST to analyze the genetic basis of the biofilm producer genes and its enhancer genes together with the antibiotic genes profile of the classical and hypervirulent.

Genomic DNA was extracted using the QIAamp micro kit (Qiagen, Hilden, Germany), agarose gel electrophoresis, and fluorometric analysis (Qubit^®^, Thermo Fisher Scientific, Waltham, MA, USA), and the isolated gDNA’s content and purity were ascertained. The purified DNA was shipped to the London School of Hygiene and Tropical Medicine (LSHTM), United Kingdom, for WGS using the Illumina MiSeq platform (QIAseq FX DNA Library Kit, QIAGEN, Hilden, Germany) with a 151 bp paired-end protocol and 100× coverage for the sequencing. CLC Genomics Workbench version 10 (CLC, Bio-QIAGEN, Aarhus, Denmark) and SPAdes version 3.530 were used to de novo assemble with reference (NCBI txid573) and quality trim the generated reads to eliminate gaps [39].

Sequence annotation was inferred by Prokka v.1.14.6 and the RAST server [40,41]. Pangenome analysis was performed using Roary with a gff annotation file produced by Prokka genome annotation [42]. The antibiotic resistance and other relevant genetic variables involved in biofilm formation, such as biofilm formation stimulation factor (*BssS* gene), toxin–antitoxin biofilm protein (*TabA*), Cyclic-di-GMP-binding biofilm dispersal mediator protein (*bdcA*), type 1 fimbriae (fimH), and *allS* (aerobactin) were then analyzed. *All* (aerobactin) is a core gene in all *K. pneumoniae* strains. The solid biofilm-forming strains possess strain-specific genes, including the *allS* gene, type III fimbriae (*mrkD, sfaS*), pili (*pilQ, ecpA*), adhesins/polysaccharides (*pgaA, pgaB, pgaC, pgaD*), CPS (*wzc, treC*), and QS (*lux*).

### 2.9. Statistical Analysis

Data analysis was conducted using Microsoft Excel and IBM SPSS Statistics version 25. Frequencies of resistant subpopulations were calculated in Excel to categorize isolates as susceptible or resistant. Further statistical analyses, including the determination of frequencies, percentages, and odds ratios, were performed in SPSS. Regression analyses and *p*-values were also calculated to assess associations and statistical significance. Ethical approval for the study was obtained from the KMU institutional review board with Ref/no: KMU/IPDM/IEC/202229, ensuring compliance with guidelines for data integrity, confidentiality, and the responsible reporting of results.

## 3. Results

Between January 2022 and June 2024, 500 samples were processed for the current investigation, of which 174 were found to be Gram-negative rods. *K. pneumoniae* was detected in 36.7% (64/174) of the total Gram-negative rods. These strains were all obtained from specimens sent to Ayub Teaching Hospital (ATH) in Abbottabad and the Mardan Medical Complex’s microbiology lab in Mardan. The identified strains were transferred to the Institute of Pathology and Diagnostic Medicine (IPDM) at KMU, Peshawar’s Microbiology Laboratory, for further processing. Following accurate identification and confirmation, the isolates underwent testing to determine their antibiotic susceptibility and the presence of various virulence factors. All 64 *K. pneumoniae* isolates were identified through biochemical testing and confirmed using the API 20E kit. The confirmed *K. pneumoniae* isolates were preserved in 30% glycerol nutrient broth at −80 °C for future use.

### 3.1. K. pneumoniae Prevalence and Distribution of Hypervirulent and Classical Klebsiella pneumoniae Strains

Of the 64 *K. pneumoniae* isolates, 26 (40.62%) were isolated from females and 38 (59.3%) from males. The distribution of isolates was as follows: urine (*n* = 22, 34.37%), pus (*n* = 19, 29.68%), blood (*n* = 13, 20.31%), fluids (*n* = 9, 14.06%), and wounds (*n* = 1, 1.56%). Hypervirulent *K. pneumoniae* (hvKp) strains were defined as hypermucoid (string positive) strains with a string test result greater than 5 mm in diameter. Of the total isolated strains, 21 (32.8%) were identified as hvKp, while the remaining 43 (67.2%) were classified as classical *K. pneumoniae* (cKp).

### 3.2. Antibiotic Resistance

**Figure 1:**
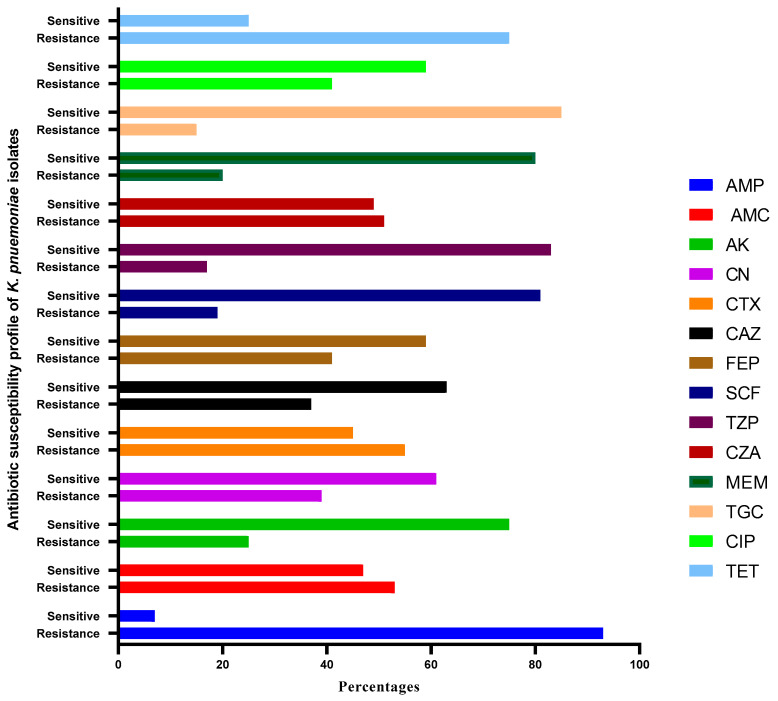
Antimicrobial susceptibility and resistance pattern of 64 *K. pneumoniae* isolates against different antibiotics. AMP—ampicillin; AK—amikacin; AMC—amoxicillin plus clavulanic acid; TZP—piperacillin plus tazobactam; CTX—cefotaxime; CAZ—ciprofloxacin; MEM—meropenem; FEP—cefepime; CZA—ceftazidime/avibactam; CN—gentamicin; CIP—ciprofloxacin; SCF—sulbactam/cefoperazone; TGC—tigecycline; TET—tetracycline.

The Kirby–Bauer disc diffusion technique results indicated that isolates exhibited the highest resistance to ampicillin (92.1%, 59/64), followed by tetracycline (75%, 48/64) and cefotaxime (54.6%, 35/64) (Table 1, Figure 1). Additionally, 42 isolates (65.6%) were multidrug-resistant (MDR), showing resistance to three classes of antibiotics. Blood isolates showed the highest resistance levels in more than three classes of antibiotics, with 32 (50%) classified as extensively drug-resistant (XDR).

**Figure 2:**
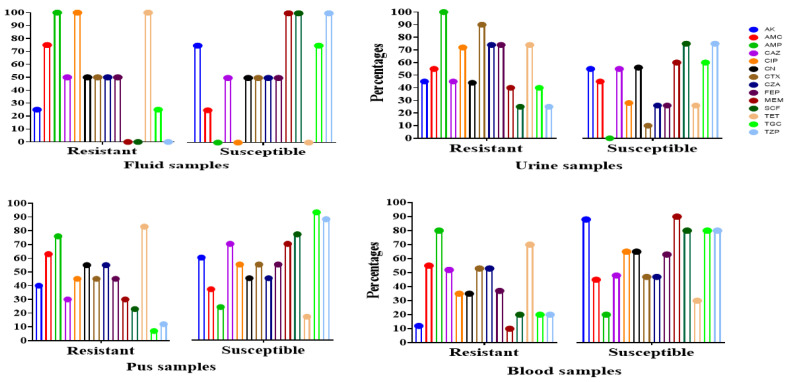
Antimicrobial susceptibility and resistance of *K. pneumoniae* isolated from clinical samples such as urine, pus, blood, and fluid. AMP—ampicillin; AK—amikacin; AMC—amoxicillin plus clavulanic acid; TZP—piperacillin plus tazobactam; CTX—cefotaxime; CAZ—ciprofloxacin; MEM—meropenem; FEP—cefepime; CZA—ceftazidime/avibactam; CN—gentamicin; CIP—ciprofloxacin; SCF—sulbactam/cefoperazone; TGC—tigecycline, TET—tetracycline.

Of the isolates examined, 42 (65.6%) were MDR. Of these, 37 (88%) showed resistance to more than three different antibiotic groups, while the remaining isolates were resistant to just three groups. MDR was present in isolates sourced from blood (12/42) and urine (12/42, 28.5%), pus (11/42, 26.1%), and fluid (5/42, 11.9%). A total of 32 (50%) isolates were XDR (Figure 2). A high percentage of cKp isolates (*n* = 43) showed resistance to ampicillin and ciprofloxacin in blood samples. All *K. pneumoniae* isolates from fluid samples showed 100% resistance to ciprofloxacin, tetracycline, and ampicillin. In total, 86% of isolates from pus samples showed resistance against tetracyclin.

### 3.3. Biofilm Production

**Figure 3:**
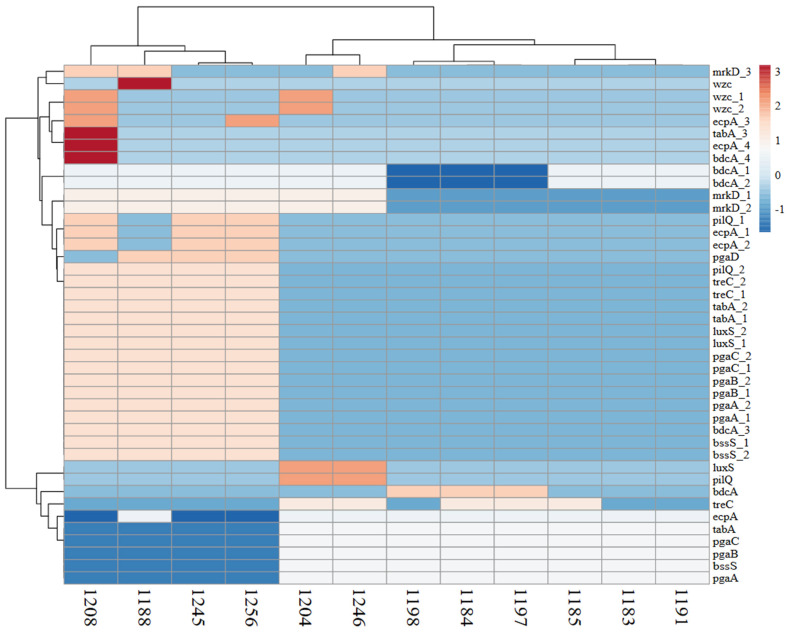
The heatmap illustrates the correlation between *K. pneumoniae* isolates (on the y-axis) and biofilm genes (on the x-axis; each row corresponds to a different gene). Biofilm producers: 1188, 1204, 1208, 1245, 1246, 1256; biofilm non-producers: 1183, 1184, 1185, 1191, 1197, 1198. The color intensity in the dendrogram indicates the degree of correlation or similarity between the different genes or gene expression profiles. The darker the color, the stronger the correlation.

In our study, only 84% (*n* = 54/64) of samples showed biofilm production (strong, moderate, and weak); the most dominant group for biofilm production was found in urine samples. Strong biofilm producers in the classical strain were found in urine and blood, whereas in hypervirulent strains, moderate biofilm producers were more than strong biofilm producers in urine samples (Figure 3). Isolates that had biofilm production were sourced from both genders (female 32/38, male 22/26) and an age range of 16–45 years (Table 2). In total, 34.3% of the *K. pneumoniae* isolates from urine samples were positive for biofilm production. A notable observation was that females exhibited a higher incidence of biofilm production, with 50% of the female samples testing positive. Additionally, individuals aged 16–45 were more likely to have stronger biofilm producers and a higher number of biofilm-producing bacteria. Urine specimens were found to have the highest odds of biofilm production compared to blood (OR = 2.93), while fluid specimens had much lower odds (OR = 0.10). The categories “46–60 years” and “>60 years” were assigned infinite odds because there were no non-producers in those age groups, making it impossible to calculate a finite odds ratio.

Ampicillin demonstrated a greater biofilm formation in antibiotic-resistant bacteria than in susceptible ones. Conversely, biofilm formation was seen in susceptible isolates, whereas resistant bacteria are non-biofilm producers and weak-to-moderate biofilm formers (Table 3).

### 3.4. Genetic Determinants of Biofilm Formation in Hypervirulent and Classical Strains

**Figure 4:**
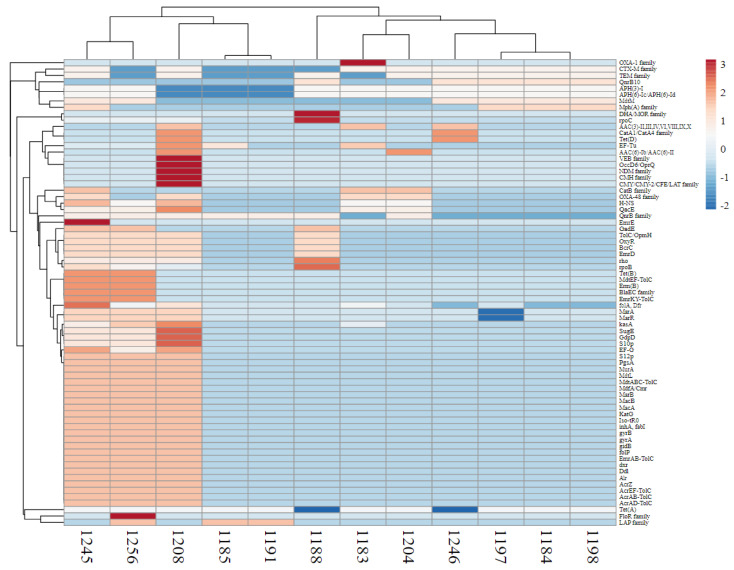
The heatmap illustrates the correlation between *K. pneumoniae* isolates (on the y-axis) and antibiotic-resistant genes (the x-axis depicts biofilm producers and non-producers; each row in the heatmap corresponds to a different resistant gene). The color intensity in the dendrogram indicates the degree of correlation or similarity between the different genes or gene expression profiles. The darker the color, the stronger the correlation.

Examining genetic variation within producers and non-producers has provided insights into biofilm production’s genetic foundation in hypervirulent and classical organisms (Figure 4). The hypervirulent producer strains (1208, 1245, and 1256) belong to ST859, ST147-1LV, and ST45-1LV, with 30, 24, and 25 producers and enhancer genes, whereas the classical strains (1188, 1204, and 1246) belong to ST1047-1LV, ST2629, and ST37 and have 24, 15, and 14 genes. The hypervirulent non-producers (1183, 1184, and 1185) belong to ST147, ST4, and ST1310, and the classical strains (1191, 1197, and 1198) belong to ST1310, ST4, and ST4 and have basic genes like *BssS, TabA,* and *bdcA* but are absent of most of the enhancing genes. *BssS*-*, TabA*-*,* and *bdcA*-like genes were present in the non-biofilm producers, but the biofilm-enhancing genes were absent. Biofilm creators possessed all enhancing genes like (1) *mrkD* genes that help to create thick biofilms of *K. pneumoniae*. Additionally, (2) increased expression of the *fimH* gene is crucial for bacterial attachment to surfaces, which promotes the creation of robust biofilms; (3) strong biofilm formation (first detected in *Klebsiella*) was significantly correlated with the expression of the *sfa* gene; (4) *pilQ* is known to be important in regulating pili function, (5) *pgaABCD* mediates bacterial species’ intercellular binding and surface adhesion; (6) *treC* has been shown to affect *K. pneumoniae* biofilm formation by regulating the production of capsular polysaccharide (CPS); and (6) *luxS* is a Type 2 quorum-sensing regulatory system that enhances the biofilm mechanism.

### 3.5. Antibiotic Resistance Gene Profile and Biofilm Producers Gene Profile

The biofilm producers’ antibiotic backgrounds were analyzed, and the results showed that the 140, 139, and 130 genes in strains 1208, 1245, and 1256 produced hypervirulent strains. In strains 1188, 1204, and 1246, the classical producers had 84, 72, and 69 genes, respectively. In contrast, the classical strains had 64, 67, and 68 (1191, 1197, and 1198), while the non-producer hypervirulent strains had 77, 68, and 67 (1183, 1184, and 1185).

**Figure 5:**
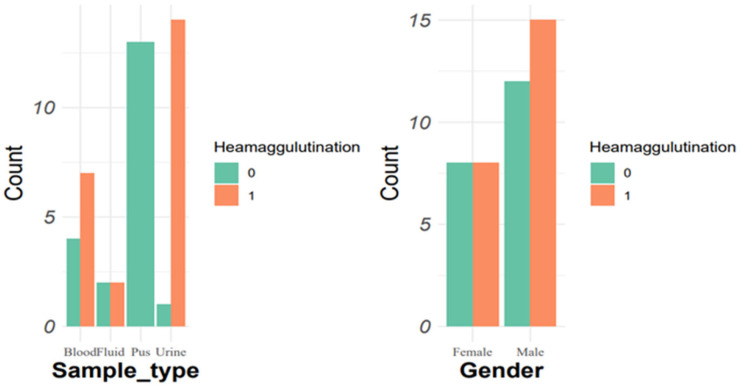
Haemagglutination assay stratified by sample types and gender in classical variants of *K. pneumoniae*.

A diverse range of resistant genes was found to be present in different isolates; for example, novobiocin resistance is linked to mutations in the *gyrA* and *gyrB* genes, which play crucial roles in the function of DNA gyrase. Resistance to beta-lactam antibiotics, such as penicillins and cephalosporins, arises due to the presence of extended-spectrum beta-lactamase (ESBL) genes, including *blaTEM*, *blaSHV*, *blaCTX-M*, *blaOXA*, and *blaKPC*. Resistance mechanisms also extend to other antibiotic classes, such as phenicols (via *floR*), macrolides (*ermB* and *mefA*), carbapenems (*blaKPC*, *blaNDM*, and *blaOXA-48*), cyclo-serine (*cycA* and *cycB*), fosmidomycin (*fosA*), fusidic acid (*fusA* and *fusB*), elfamycins (*rpoB*), deaminopyrimidines like trimethoprim (*dfrA* and *dfrB*), sulfonamides (*sul1* and *sul2*), fluoroquinolones (*gyrA*, *gyrB*, *parC*, and *parE*), quinolones (*gyrA* and *gyrB*), bicyclomycins (*bcyA* gene), rifamycins (*rpoB*), peptide antibiotics such as vancomycin (*vanA*, *vanB*, and *bacA*), lincosamides (*erm*), streptogramins (*erm*, *vgaA*, and *vgaB*), isoniazid (*inhA*, *katG*, and *ahpC*), ethionamide (*ethA*), triclosan (*fabI*), nucleoside antibiotics (e.g., *pyre*), and glycylines (*tet(X)*). The screened antibiotics genes are mentioned in (Figure 5).

### 3.6. Hemagglutination Assay

**Figure 6:**
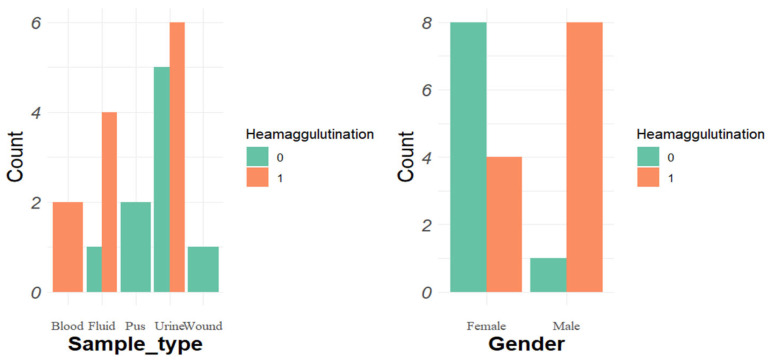
Haemagglutination assay stratified by sample types and gender in hypervirulent *K. pneumoniae.*.

Out of the total isolated strains, 34 (53%) were positive, while 30 (47%) were negative in the hemagglutination assay. Among hvKp, 57% (*n* = 12) were positive for hemagglutination, while cKp showed a 43.4% (*n* = 23) positivity rate for hemagglutination (Figure 6). In cKp, the hemagglutination ability was high in urine isolates, i.e., 93.3% (*n* = 14/15), followed by blood 63.6% (*n* = 7/11), fluid 50% (*n* = 2/4), and pus samples 0% (*n* = 0/13). In hvKp, the highest percentage was found in urine samples, 83.3% (*n* = 5/6), followed by fluids, 66.6% (*n* = 4/6), blood, 60% (*n* = 3/5), whereas pus showed a 0% rate.

### 3.7. Hemolysis Assay

None of our samples showed any hemolysin activity on blood agar.

## 4. Discussion

An increasingly common pathotype of the bacteria *K. pneumoniae* is hypervirulent, which is clinically distinguished by its capacity to infect both immunocompetent and healthy people with potentially fatal infections, so this study identified significant findings with critical implications for both clinical practice and public health. *Klebsiella* spp. accounted for 36.7% of the identified Gram-negative bacteria in this investigation, similar to previous reports [43,44,45,46,47]. The differences in prevalence rates seen in other studies might be caused by various factors, including research location, collection type, seasonal change, and environmental parameters, including the study site’s pH level, temperature, and humidity [44].

We observed that the male population is highly affected, consistent with other studies [48]. Men consume more alcohol and smoke more than women do, which may be a contributing factor in their increased risk of contracting *K. pneumoniae* infections [48]. Sixty-four *K. pneumoniae* strains were recovered from various samples, with 33% hvKp, which falls between the rates of similar studies (15.8% [49] and 64% [50]). The mean age ± SD of those diagnosed with hvKp was 31.43 ± 21.5 years and 30.33 ± 14.92 among cKp, comparable to previous works [49], but other studies have found that the prevalence of hvKp was widespread in elderly patients [50,51]. The majority of identified *Klebsiella*, including cKp and hvKp, had antibiotic resistance to ampicillin (92.1%), tetracycline (75%), and cefotaxime (54.6%). Other studies found that most bacteria were resistant to trimethoprim–sulfamethoxazole, ciprofloxacin, and nitrofurantoin [52].

The WHO considers carbapenem-resistant *K. pneumoniae* to be a “serious cause for concern.” Compared to the previously published 62% and 48% meropenem resistance rates [50,52,53], the current study’s results for hvKp meropenem resistance rates were 14% and 20% for the classical variants. This discrepancy may be due to the patients’ increased use of the antibiotic as well as the isolation of samples from hospitalized patients. When comparing their antibiotic resistance profiles, we observed a substantial difference in antibiotic resistance between hvKp and cKp.

Following reports from the USA, MDR *K. pneumoniae* was later seen in Europe, South America, and Asia [54]. Because there are few efficient antibiotics on hand, managing AMR in MDR *K. pneumoniae* is an essential challenge for doctors and can lead to higher death rates, prolonged hospital stays, and excessive treatment expenditures [54]. Our analysis demonstrated that it had the highest resistance to ampicillin, followed by tetracycline and cefotaxime, with a reported XDR resistance of 50% and MDR resistance of 65.6%. Numerous studies have reported a growing trend of antibiotic resistance and the emergence of multidrug-resistant (MDR) *K. pneumoniae* [13,55,56,57,58].

The estimated global prevalence of nosocomial MDR *K. pneumoniae* was 32.8%, but there is heterogeneity between regions [59], with South America with 72.4% and North America with 12.9%. There is a heterogeneity of MDR *K. pneumoniae* prevalence at a country level, with Brazilian (61.9%) [60], Bangladeshi (82%) [61], and Egyptian hospitals (77.7%) [62] having high rates. In total, 59.3% of the isolates were ESBL-positive, and 85.7% of the MDR isolates had this resistance mechanism, similar to results found in a recent study in Poland [63]. This problem has become a serious worry for healthcare systems and a life-threatening issue, emphasizing the need to adopt hospital antimicrobial stewardship (AMS) programs to encourage the prudent use of antibiotics.

In our study, the percentage of *K. pneumoniae* isolates that produced ESBLs was lowest among fluid isolates (1.5%) and largest among isolates from urine, followed by blood and pus. *Klebsiella* spp. is becoming resistant to carbapenems at a drastic rate, even though they represent the last line of defense for isolates expressing ESBL and *AmpC* [64]. In our study, 20% of *Klebsiella* spp. strains were resistant to meropenem. The distribution of CRKp isolates revealed the highest prevalence in pus samples, followed sequentially by blood and urine, and the lowest in fluid specimens. This pattern diverges from previous reports, identifying sputum as the primary source of CRKp isolation, followed by urine and blood [65].

Our results showed that a large proportion (84.3%) of *K. pneumoniae* isolates were biofilm producers, inconsistent with the report that also reported high biofilm producers of *K. pneumoniae* [66]. The *K. pneumoniae* isolates with biofilm-forming potential can cause other serious consequences, such as implant-associated infections [67] and antibiotic resistance, and pose a risk for nosocomial opportunistic infections [68,69].

The genetic processes for adhesion (fimbriae and pili), cohesion (adhesins and polysaccharides), CPS, and QS are known to be involved in successful biofilm formation in *K. pneumoniae* [70,71,72]. Extensive research has demonstrated that type 3 fimbriae is pivotal in biofilm formation, particularly in *K. pneumoniae* strains. This is likely attributable to its capacity to mediate surface adhesion, facilitating the colonization and clonal proliferation of the bacteria [73,74,75].

Our research found a strong correlation between the presence of the *luxS, mrkA, pgaA*, and *wzm* genes and biofilm formation in *K. pneumoniae* isolates. The presence of *luxS, mrkA, pgaA*, and *wzm* genes was notably more frequent in biofilm-producing *K. pneumoniae* isolates than in non-biofilm-producing strains. Consistent with the findings of our study, Mirzaie et al. observed that the prevalence of biofilm-associated genes, including *mrkA, fimH*, and *mrkD*, was *K. pneumoniae* isolates [11]. Another study reported the presence of *fimH, mrkA*, and *mrkD* in 87.5%, 46.4%, and 53.6% of *K. pneumoniae* isolates, respectively [76]. Shadkam and colleagues, in their investigation of biofilm formation genes, found that 98% of isolates carried the *luxS* gene, 96% contained the *treC* gene, and 34% harbored the *wza* gene [77]. Our research corroborates the significant role of several genes, including *luxS, mrkA, pgaA*, and *wzm*, in *K. pneumoniae* biofilm formation, as previously highlighted in other studies [72,78,79].

Nevertheless, the *MrkD* protein at the tip of the fimbriae confers the adhesive characteristic for practical attachment, aiding in improved adhesion to the basolateral surfaces in vivo, such as bronchial or urinary tract epithelia [80]. In *K. pneumoniae*, pili function is known to be regulated by the *E. Coli* adhesive structures *pilQ* and common pilus (*ecp*). The current investigation found *EcpA* and *pilQ* in practically all of the *K. pneumoniae* isolates.

Hemagglutination test results were positive for 35 (54.6%) isolated strains. HvKp exhibits 57% (*n* = 12) positivity for hemagglutination, whereas cKp exhibited 53.4% (*n* = 23) positivity. A recent study found a higher total (67%) of isolated *K. pneumoniae* positive for hemagglutination [81]. The analysis of hemolysis revealed 0%, which contrasts with other studies (8% [82], 20% [83]).

## 5. Conclusions

This study demonstrates the presence of hypervirulent *K. pneumoniae* (hvKp) strains in clinical samples collected from various sites in Pakistan. The findings highlight a higher prevalence of hvKp in younger patients, contrasting with other studies reporting its prevalence among elderly patients. Additionally, the study underscores the considerable antibiotic resistance observed in both classical and hypervirulent strains, particularly against ampicillin, tetracycline, and cefotaxime. The presence of various virulence-associated and biofilm-enhancing genes, such as *mrkD****,*** *pgaABCD****,*** *fimH****,*** *treC****,*** *wzc****,*** *pilQ*, and *luxS*, in hypervirulent strains further highlights the potential for increased morbidity and mortality in affected patients. The findings also reveal a concerning overlap between MDR, XDR strains, and virulence factors, which is particularly alarming. While the findings provide valuable insights, limitations such as the small sample size and limited geographic coverage must be acknowledged. Future research should explore larger, multicenter studies to understand resistance and virulence dynamics better, as well as develop targeted diagnostic and treatment strategies. Continuous surveillance and effective management remain essential to mitigate the public health impact of these infections.

## Figures and Tables

**Table 1:** Antibiotic susceptibility profile of *K. pneumoniae* (*n* = 64).

Antibiotic	Resistance *n* (%)	Intermediate *n* (%)
Ampicillin (AMP)	59 (92.1)	None
Amoxicillin-clavulanic acid (AMC)	34 (53.1)	0
Amikacin (AK)	16 (25.0)	0
Gentamicin (CN)	25 (39.0)	0
Cefotaxime (CTX)	35 (54.6)	0
Ceftazidime (CAZ)	24 (37.5)	0
Cefepime (FEP)	26 (40.6)	0
Sulbactam/Cefoperazone (SCF)	12 (18.7)	0
Piperacillin–tazobactam (TZP)	11 (17.1)	0
Ceftazidime/avibactam (CZA)	33 (51.5)	0
Meropenem (MEM)	13 (20.3)	0
Tigecycline (TGC)	10 (15.6)	0
Ciprofloxacin (CIP)	26 (40.6)	0
Tetracycline (TET)	48 (75.0)	1 (1.56)
MDR total 42		
XDR total 32		

**Table 2:** Biofilm production among *K. pneumoniae* isolates considering clinical specimens, gender, and age with odds and odd ratios.

Character	Strong *n*(%)	Moderate *n* (%)	Weak *n* (%)	Total Producer *n* (%)	Non-Producer *n* (%)	Odds	OR vs. Blood	OR vs. Urine	OR vs. Male	OR vs. Female
Clinical specimen										
Blood	7 (41.1)	3 (17.6)	5 (29.4)	15 (88.2)	2 (11.7)	7.5	1	0.34	1.36	1.41
Fluid	0 (0)	2 (28.5)	1 (14.2)	3 (42.8)	4 (57.1)	0.75	0.1	0.03	0.14	0.14
Pus	5 (29.4)	6 (35.2)	3 (17.6)	14 (82.3)	3 (17.6)	4.67	0.62	0.21	0.85	0.88
Urine	10 (43.4)	7 (30.4)	5 (21.7)	22 (95.6)	1 (4.3)	22	2.93	1	4	4.13
Gender										
Male	8 (30.7)	8 (30.7)	6 (23)	22 (84.6)	4 (15.3)	5.5	0.73	0.25	1	1.03
Female	14 (36.8)	10 (26.3)	8 (21)	32 (84.2)	6 (15.7)	5.33	0.71	0.24	0.97	1
Age group										
0–15 years	3 (37.5)	1 (12.5)	1 (12.5)	5 (62.5)	3 (37.5)	1.67	0.22	0.08	0.3	0.31
16–45 years	16 (32)	16 (32)	11 (22)	43 (86)		6.14	0.82	0.28	1.12	1.15
46–60 years	2 (50)	1 (25)	1 (25)	4 (100)	0 (0)	infinite	infinite	Infinite	Infinite	Infinite
>60 years	1 (50)	0 (0)	1 (50)	2 (100)	0 (0)	infinite	infinite	infinite	infinite	Infinite

**Table 3:** Comparison of biofilm formation in antibiotic-resistant *K. pneumoniae* strains.

Antibiotics	AST Pattern		Strength of Biofilm Formation				Odd Ratios
		Number	Non-Producer	Poor	Moderate	Strong	
AK	R	15	2	4	3	6	1.27
AK	S	49	8	10	15	16	
AMC	R	32	8	7	9	8	0.20
AMC	S	32	2	7	9	14	
AMP	R	57	10	14	15	18	Infinite
AMP	S	7	0	0	3	4	
CAZ	R	24	3	4	8	9	1.49
CAZ	S	40	7	10	10	13	
CIP	R	26	6	6	6	8	0.39
CIP	S	38	4	8	12	14	
CN	R	25	3	7	6	9	1.60
CN	S	39	7	7	12	13	
CTX	R	36	4	8	10	14	2.18
CTX	S	28	6	6	8	8	
CZA	R	33	5	8	9	11	1.08
CZA	S	31	5	6	9	11	
FEP	R	25	4	6	7	8	0.95
FEP	S	39	6	8	12	13	
MEM	R	12	3	3	1	5	0.47
MEM	S	52	7	11	17	17	
SCF	R	12	2	4	3	3	0.91
SCF	S	52	8	10	15	19	
TET	I	1	0	0	0	1	Infinite
TET	R	47	9	11	12	15	0.28
TET	S	16	1	3	6	6	
TGC	R	10	1	2	4	3	1.80
TGC	S	54	9	12	14	19	
TZP	R	11	3	3	2	3	0.41
TZP	S	53	7	11	16	19	

## Data Availability

1183_contigs with accession number (AC) SAMN43092440, 1184_contigs AC SAMN43092441, 1185_contigs AC SAMN43092442, 1188_contigs AC SAMN43092443, 1191_contigs AC SAMN43092445, 1197_contigs AC SAMN43092450, 1198_contigs AC SAMN43092451, 1204_contigs AC SAMN43092454, 1208_contigs AC SAMN43092455, 1245_contigs AC SAMN43092461, 1246_contigs AC SAMN43092462, 1256_contigs AC SAMN43092469.

## References

[1] Podschun R., Ullmann U. (1998). Klebsiella spp. as nosocomial pathogens: Epidemiology, taxonomy, typing methods, and pathogenicity factors. Clin. Microbiol. Rev..

[2] Bagley S.T. (1985). Habitat association of Klebsiella species. Infect. Control. Hosp. Epidemiol..

[3] Vuotto C., Longo F., Pascolini C., Donelli G., Balice M.P., Libori M., Tiracchia V., Salvia A., Varaldo P.E. (2017). Biofilm formation and antibiotic resistance in *Klebsiella pneumoniae* urinary strains. J. Appl. Microbiol..

[4] Ghashghaee A., Behzadifar M., Azari S., Farhadi Z., Bragazzi N.L., Behzadifar M., Shahri S.S.S., Ghaemmohamadi M.S., Ebadi F., Mohammadibakhsh R. (2018). Prevalence of nosocomial infections in Iran: A systematic review and meta-analysis. Med. J. Islam. Repub. Iran..

[5] Ashayeri-Panah M., Feizabadi M.M., Eftekhar F. (2014). Correlation of multi-drug resistance, integron and blaESBL gene carriage with genetic fingerprints of extended-spectrum β-lactamase producing *Klebsiella pneumoniae*. Jundishapur J. Microbiol..

[6] Chew K.L., Lin R.T.P., Teo J.W. (2017). *Klebsiella pneumoniae* in Singapore: Hypervirulent infections and the carbapenemase threat. Front. Cell. Infect. Microbiol..

[7] Zhang Y., Yao Z., Zhan S., Yang Z., Wei D., Zhang J., Li J., Kyaw M.H. (2014). Disease burden of intensive care unit-acquired pneumonia in China: A systematic review and meta-analysis. Int. J. Infect. Dis..

[8] Zhang Y., Wang Q., Yin Y., Chen H., Jin L., Gu B., Xie L., Yang C., Ma X., Li H. (2018). Epidemiology of carbapenem-resistant Enterobacteriaceae infections: Report from the China CRE Network. Antimicrob. Agents Chemother..

[9] Cubero M., Marti S., Domínguez M., González-Díaz A., Berbel D., Ardanuy C. (2019). Hypervirulent *Klebsiella pneumoniae* serotype K1 clinical isolates form robust biofilms at the air-liquid interface. PLoS ONE.

[10] Victor L.Y., Hansen D.S., Ko W.C., Sagnimeni A., Klugman K.P., Von Gottberg A., Goossens H., Wagener M.M., Benedi V.J., International Klebsiella Study Group (2007). Virulence characteristics of Klebsiella and clinical manifestations of *K. pneumoniae* bloodstream infections. Emerg. Infect. Dis..

[11] Mirzaie A., Ranjbar R. (2021). Antibiotic resistance, virulence-associated genes analysis and molecular typing of *Klebsiella pneumoniae* strains recovered from clinical samples. AMB Express.

[12] Yousefi B., Abdolshahi A., Dadashpour M., Pahlevan D., Ghaffari H., Eslami M. (2023). Evaluation of genes involved in the binding and invasion of *Klebsiella pneumoniae* including fimH-1, entB, iutA, rmpA and cnf-1 genes in patients with urinary tract infection. Rev. Med. Microbiol..

[13] Kot B., Piechota M., Szweda P., Mitrus J., Wicha J., Grużewska A., Witeska M. (2023). Virulence analysis and antibiotic resistance of *Klebsiella pneumoniae* isolates from hospitalised patients in Poland. Sci. Rep..

[14] Martin R.M., Bachman M. (2018). Colonization, infection, and the accessory genome of *Klebsiella pneumoniae*. Front. Cell. Infect. Microbiol..

[15] Navon-Venezia S., Kondratyeva K., Carattoli A. (2017). *Klebsiella pneumoniae*: A major worldwide source and shuttle for antibiotic resistance. FEMS Microbiol. Rev..

[16] Xu J., Zhao Z., Ge Y., He F. (2020). Resistance D. Rapid emergence of a pandrug-resistant *Klebsiella pneumoniae* ST11 isolate in an inpatient in a teaching hospital in China after treatment with multiple broad-spectrum antibiotics. Infect. Drug Resist..

[17] Fatima S., Liaqat F., Akbar A., Sahfee M., Samad A., Anwar M., Iqbal S., Khan S.A., Sadia H., Makai G. (2021). Virulent and multidrug-resistant *Klebsiella pneumoniae* from clinical samples in Balochistan. Int. Wound J..

[18] Saddam S., Khan M., Jamal M., Rehman S.U., Slama P., Horky P. (2023). Multidrug resistant *Klebsiella pneumoniae* reservoir and their capsular resistance genes in cow farms of district Peshawar, Pakistan. PLoS ONE.

[19] Haider M.H., McHugh T.D., Roulston K., Arruda L.B., Sadouki Z., Riaz S. (2022). Detection of carbapenemases bla OXA48-bla KPC-bla NDM-bla VIM and extended-spectrum-β-lactamase bla OXA1-bla SHV-bla TEM genes in Gram-negative bacterial isolates from ICU burns patients. Ann. Clin. Microbiol. Antimicrob..

[20] Chaudhry T.H., Aslam B., Arshad M.I., Alvi R.F., Muzammil S., Yasmeen N., Aslam M.A., Khurshid M., Rasool M.H., Baloch Z. (2020). Emergence of bla NDM-1 Harboring *Klebsiella pneumoniae* ST29 and ST11 in Veterinary Settings and Waste of Pakistan. Infect. Drug Resist..

[21] Parveen S., Saqib S., Ahmed A., Shahzad A., Ahmed N. (2020). Prevalence of MRSA colonization among healthcare-workers and effectiveness of decolonization regimen in ICU of a Tertiary care Hospital, Lahore, Pakistan. Adv. Life Sci..

[22] Bashir N., Dablool A.S., Khan M.I., Almalki M.G., Ahmed A., Mir M.A., Hamdoon A.A.E., Elawad M.A., Mosa O.F., Niyazov L.N. (2023). Antibiotics resistance as a major public health concern: A pharmaco-epidemiological study to evaluate prevalence and antibiotics susceptibility-resistance pattern of bacterial isolates from multiple teaching hospitals. J. Infect. Public Health.

[23] Rahman S., Hameed A., Roghani M., Ullah Z. (2002). Multidrug resistant neonatal sepsis in Peshawar, Pakistan. Arch. Dis. Child. Fetal Neonatal Ed..

[24] Bilal H., Khan M.N., Rehman T., Hameed M.F., Yang X. (2021). Antibiotic resistance in Pakistan: A systematic review of past decade. BMC Infect. Dis..

[25] Ahmed N., Ali Z., Riaz M., Zeshan B., Wattoo J.I., Aslam M.N. (2020). Evaluation of antibiotic resistance and virulence genes among clinical isolates of Pseudomonas aeruginosa from cancer patients. Asian Pac. J. Cancer Prev..

[26] Handbook of Specimen Collection and Handling in Microbiology. https://stacks.cdc.gov/view/cdc/7700/cdc_7700_DS1.pdf.

[27] Akbar A., Anal A.K. (2014). Occurrence of *Staphylococcus aureus* and evaluation of anti-staphylococcal activity of *Lactococcus lactis* subsp. lactis in ready-to-eat poultry meat. Ann. Microbiol..

[28] Cheesbrough M. (2006). District Laboratory Practice in Tropical Countries, Part 2.

[29] (2017). Performance Standards for Antimicrobial Susceptibility Testing.

[30] (2020). Performance Standards for Antimicrobial Susceptibility Testing A CLSI Supplement for Global Application. Performance Standards for Antimicrobial Susceptibility Testing Performance Standards for Antimicrobial Susceptibility Testing.

[31] Basak S., Singh P., Rajurkar M. (2016). Multidrug resistant and extensively drug resistant bacteria: A study. J. Pathog..

[32] Ono R., Kitagawa I. (2021). Positive string test in a patient with hypermucoviscous *Klebsiella pneumoniae* infection. QJM Int. J. Med..

[33] McFarland Standards. https://en.wikipedia.org/wiki/McFarland_standards.

[34] Rosenthal L. (1943). Agglutinating properties of *Escherichia coli*: Agglutination of erythrocytes, leucocytes, thrombocytes, spermatozoa, spores of molds, and pollen by strains of *E. coli*. J. Bacteriol..

[35] Neter E. (1956). Bacterial hemagglutination and hemolysis. Bacteriol. Rev..

[36] Hussein A.Q., Al-Meani S.A. (2022). Assessment the relationship of Antibiotic profile and virulence factors in *Klebsiella pneumoniae*. Int. J. Health Sci..

[37] Makia R.S., Fadhil A.M., Ismail M.C. (2013). Biofilm production as a virulence factor in Uropathogenic bacteria and yeasts. J. Biotechnol. Res. Cent..

[38] Kowalska J., Maćkiw E., Stasiak M., Kucharek K., Postupolski J. (2020). Biofilm-forming ability of pathogenic bacteria isolated from retail food in Poland. J. Food Prot..

[39] Bankevich A., Nurk S., Antipov D., Gurevich A.A., Dvorkin M., Kulikov A.S., Lesin V.M., Nikolenko S.I., Pham S., Prjibelski A.D. (2012). SPAdes: A new genome assembly algorithm and its applications to single-cell sequencing. J. Comput. Biol..

[40] Aziz R.K., Bartels D., Best A.A., DeJongh M., Disz T., Edwards R.A., Formsma K., Gerdes S., Glass E.M., Kubal M. (2008). The RAST Server: Rapid annotations using subsystems technology. BMC Genom..

[41] Seemann T. (2014). Prokka: Rapid prokaryotic genome annotation. Bioinformatics.

[42] Page A.J., Cummins C.A., Hunt M., Wong V.K., Reuter S., Holden M.T., Fookes M., Falush D., Keane J.A., Parkhill J. (2015). Roary: Rapid large-scale prokaryote pan genome analysis. Bioinformatics.

[43] Kayastha K., Dhungel B., Karki S., Adhikari B., Banjara M.R., Rijal K.R., Ghimire P. (2020). Extended-spectrum β-lactamase-producing *Escherichia coli* and Klebsiella species in pediatric patients visiting International Friendship Children’s Hospital, Kathmandu, Nepal. Infect. Dis. Res. Treat..

[44] Dhungana K., Awal B.K., Dhungel B., Sharma S., Banjara M.R., Rijal K.R. (2019). Detection of *Klebsiella pneumoniae* carbapenemase (KPC) and metallo betalactamae (MBL) producing Gram negative bacteria isolated from different clinical samples in a Transplant Center, Kathmandu, Nepal. ASMI.

[45] Poudyal S., Bhatta D.R., Shakya G., Upadhyaya B., Dumre S.P., Buda G., Kandel B.P. (2011). Extended spectrum â-lactamase producing multidrug resistant clinical bacterial isolates at National Public Health Laboratory, Nepal. Med. Coll. J..

[46] Bina M., Pournajaf A., Mirkalantari S., Talebi M., Irajian G. (2015). Detection of the *Klebsiella pneumoniae* carbapenemase (KPC) in *K. pneumoniae* Isolated from the Clinical Samples by the Phenotypic and Genotypic Methods. Iran. J. Pathol..

[47] Sah R.S.P., Dhungel B., Yadav B.K., Adhikari N., Thapa Shrestha U., Lekhak B., Banjara M.R., Adhikari B., Ghimire P., Rijal K.R. (2021). Detection of TEM and CTX-M Genes in *Escherichia coli* isolated from clinical specimens at tertiary care heart hospital, Kathmandu, Nepal. Diseases.

[48] Nirwati H., Sinanjung K., Fahrunissa F., Wijaya F., Napitupulu S., Hati V.P., Hakim M.S., Meliala A., Aman A.T., Nuryastuti T. (2019). Biofilm formation and antibiotic resistance of *Klebsiella pneumoniae* isolated from clinical samples in a tertiary care hospital, Klaten, Indonesia. BMC Proc..

[49] Alharbi M.T., Almuhayawi M.S., Nagshabandi M.K., Tarabulsi M.K., Alruhaili M.H., Gattan H.S., Al Jaouni S.K., Selim S., Alanazi A., Alruwaili Y. (2023). Antimicrobial resistance pattern, pathogenicity and molecular properties of hypervirulent Klebsiella pneumonia (hvKp) among hospital-acquired infections in the intensive care unit (ICU). Microorganisms.

[50] Mohamed Hassan N.A., Badawy Dawood A.H., Mohamed M.F., Sayed S.A., Mohamed D.H. (2024). Distribution, characterization and antibiotic resistance of hypervirulent *Klebsiella pneumoniae* (hvKp) strains versus classical strains (CKp) causing healthcare associated infections in Sohag University Hospitals. Microbes Infect. Dis..

[51] Li L., Li S., Wei X., Lu Z., Qin X., Li M., Saad H.A., Tawab A.A. (2023). Infection with Carbapenem-resistant Hypervirulent *Klebsiella pneumoniae*: Clinical, virulence and molecular epidemiological characteristics. Antimicrob. Resist. Infect. Control..

[52] Osama D.M., Zaki B.M., Khalaf W.S., Mohamed M.Y.A., Tawfick M.M., Amin H.M. (2023). Occurrence and molecular study of hypermucoviscous/hypervirulence trait in gut commensal *K. pneumoniae* from healthy subjects. Microorganisms.

[53] Elmonir W., Abd El-Aziz N.K., Tartor Y.H., Moustafa S.M., Abo Remela E.M., Eissa R., Saad H.A., Tawab A.A. (2021). Emergence of colistin and carbapenem resistance in extended-spectrum β-lactamase producing *Klebsiella pneumoniae* isolated from chickens and humans in Egypt. Biology.

[54] Hou X., Song X., Ma X., Zhang S., Zhang J. (2015). Molecular characterization of multidrug-resistant *Klebsiella pneumoniae* isolates. Microbiology.

[55] Sharma A., Thakur A., Thakur N., Kumar V., Chauhan A., Bhardwaj N. (2023). Changing trend in the antibiotic resistance pattern of Klebsiella pneumonia isolated from endotracheal aspirate samples of ICU patients of a tertiary care hospital in North India. Cureus.

[56] Jalal N.A., Al-Ghamdi A.M., Momenah A.M., Ashgar S.S., Bantun F., Bahwerth F.S., Hariri S.H., Johargy A.K., Barhameen A.A., Al-Said H.M. (2023). Prevalence and antibiogram pattern of *Klebsiella pneumoniae* in a tertiary care hospital in makkah, Saudi Arabia: An 11-year experience. Antibiotics.

[57] Esfahani S.N.M., Rostami S., Astaraki A. (2024). Antibiotic Susceptibility of Klebsiella pneumonia Isolates from Hospitalized Patients in Three Referral Medical Centers in Isfahan, Iran. Zahedan J. Res. Med. Sci..

[58] Awoke T., Teka B., Seman A., Sebre S., Yeshitela B., Aseffa A., Mihret A., Abebe T. (2021). High prevalence of multidrug-resistant *Klebsiella pneumoniae* in a tertiary care hospital in Ethiopia. Antibiotics.

[59] Mohd Asri N.A., Ahmad S., Mohamud R., Mohd Hanafi N., Mohd Zaidi N.F., Irekeola A.A., Shueb R.H., Yee L.C., Noor N.M., Mustafa F.H. (2021). Global prevalence of nosocomial multidrug-resistant *Klebsiella pneumoniae*: A systematic review and meta-analysis. Antibiotics.

[60] Nakamura-Silva R., Cerdeira L., Oliveira-Silva M., da Costa K.R.C., Sano E., Fuga B., Moura Q., Esposito F., Lincopan N., Wyres K. (2022). Multidrug-resistant *Klebsiella pneumoniae*: A retrospective study in Manaus, Brazil. Arch. Microbiol..

[61] Aminul P., Anwar S., Molla M.A., Miah R.A. (2021). Evaluation of antibiotic resistance patterns in clinical isolates of *Klebsiella pneumoniae* in Bangladesh. Biosaf. Health.

[62] Wasfi R., Elkhatib W.F., Ashour H.M. (2016). Molecular typing and virulence analysis of multidrug resistant *Klebsiella pneumoniae* clinical isolates recovered from Egyptian hospitals. Sci. Rep..

[63] Sękowska A., Chudy M., Gospodarek-Komkowska E. (2020). Emergence of colistin-resistant *Klebsiella pneumoniae* in Poland. Acta Microbiol. Immunol. Hung..

[64] Meletis G. (2016). Carbapenem resistance: Overview of the problem and future perspectives. Ther. Adv. Infect. Dis..

[65] Gandor N.H.M., Amr G.E.-S., Eldin Algammal S.M.S., Ahmed A.A. (2022). Characterization of carbapenem-resistant *K. pneumoniae* isolated from intensive care units of zagazig university hospitals. Antibiotics.

[66] Gao X., Wang H., Wu Z., Sun P., Yu W., Chen D., Mao Y., Fang L., Qian J., Li L. (2024). The Characteristic of Biofilm Formation in ESBL-Producing *K. pneumoniae* Isolates. Can. J. Infect. Dis. Med. Microbiol..

[67] Bjarnsholt T. (2013). The role of bacterial biofilms in chronic infections. APMIS.

[68] Borges A., JSaavedra M., Simoes M. (2015). Insights on antimicrobial resistance, biofilms and the use of phytochemicals as new antimicrobial agents. Curr. Med. Chem..

[69] Yang D., Zhang Z. (2008). Biofilm-forming *Klebsiella pneumoniae* strains have greater likelihood of producing extended-spectrum β-lactamases. J. Hosp. Infect..

[70] Alcántar-Curiel M.D., Blackburn D., Saldaña Z., Gayosso-Vázquez C., Iovine N., De la Cruz M.A., Girón J.A. (2013). Multi-functional analysis of *Klebsiella pneumoniae* fimbrial types in adherence and biofilm formation. Virulence.

[71] Wu M.-C., Lin T.-L., Hsieh P.-F., Yang H.-C., Wang J.-T. (2011). Isolation of genes involved in biofilm formation of a *Klebsiella pneumoniae* strain causing pyogenic liver abscess. PLoS ONE.

[72] Chen L., Wilksch J.J., Liu H., Zhang X., Torres V.V., Bi W., Mandela E., Cao J., Li J., Lithgow T. (2020). Investigation of LuxS-mediated quorum sensing in *Klebsiella pneumoniae*. J. Med. Microbiol..

[73] Di Martino P., Cafferini N., Joly B., Darfeuille-Michaud A. (2003). *Klebsiella pneumoniae* type 3 pili facilitate adherence and biofilm formation on abiotic surfaces. Res. Microbiol..

[74] Stahlhut S.G., Struve C., Krogfelt K.A., Reisner A. (2012). Biofilm formation of *Klebsiella pneumoniae* on urethral catheters requires either type 1 or type 3 fimbriae. FEMS Immunol. Med. Microbiol..

[75] Huang Y.-J., Liao H.-W., Wu C.-C., Peng H.-L. (2009). MrkF is a component of type 3 fimbriae in *Klebsiella pneumoniae*. Res. Microbiol..

[76] Makhrmash J., Al-Aidy S., Qaddoori B. (2022). Investigation of biofilm virulence genes prevalence in *Klebsiella pneumoniae* isolated from the urinary tract infections. Arch. Razi Inst..

[77] Shadkam S., Goli H.R., Mirzaei B., Gholami M., Ahanjan M. (2021). Correlation between antimicrobial resistance and biofilm formation capability among *Klebsiella pneumoniae* strains isolated from hospitalized patients in Iran. Ann. Clin. Microbiol. Antimicrob..

[78] Li Y., Ni M. (2023). Regulation of biofilm formation in *Klebsiella pneumoniae*. Front. Microbiol..

[79] Fang R., Liu H., Zhang X., Dong G., Li J., Tian X., Wu Z., Zhou J., Cao J., Zhou T. (2021). Difference in biofilm formation between carbapenem-resistant and carbapenem-sensitive *Klebsiella pneumoniae* based on analysis of mrkH distribution. Microb. Pathog..

[80] Murphy C.N., Clegg S. (2012). *Klebsiella pneumoniae* and type 3 fimbriae: Nosocomial infection, regulation and biofilm formation. Future Microbiol..

[81] Zafar S., Hanif S., Akhtar H., Faryal R. (2019). Emergence of hypervirulent *K. pneumoniae* causing complicated UTI in kidney stone patients. Microb. Pathog..

[82] Imtiaz W., Syed Z., Rafaque Z., Andrews S.C., Dasti J.I. (2021). Analysis of antibiotic resistance and virulence traits (genetic and phenotypic) in *Klebsiella pneumoniae* clinical isolates from Pakistan: Identification of significant levels of carbapenem and colistin resistance. Infect. Drug Resist..

[83] Dougnon V., Assogba P., Mohammed J., Agbankpe J., Deguenon E., Fabiyi K., Dougnon J., Baba-Moussa L., Bankole H. (2021). Urinary tract infections in Benin: Exploring the virulence factors and antibiotic resistance and virulence genes among bacterial isolates. Int. J. Pathog. Res..

